# Measurement of Cerebral White Matter Perfusion Using Pseudocontinuous Arterial Spin Labeling 3T Magnetic Resonance Imaging – an Experimental and Theoretical Investigation of Feasibility

**DOI:** 10.1371/journal.pone.0082679

**Published:** 2013-12-06

**Authors:** Wen-Chau Wu, Shu-Chi Lin, Danny J. Wang, Kuan-Lin Chen, Ying-Ding Li

**Affiliations:** 1 Graduate Institute of Oncology, National Taiwan University, Taipei, Taiwan; 2 Graduate Institute of Biomedical Electronics and Bioinformatics, National Taiwan University, Taipei, Taiwan; 3 Graduate Institute of Clinical Medicine, National Taiwan University, Taipei, Taiwan; 4 Department of Medical Imaging, National Taiwan University Hospital, Taipei, Taiwan; 5 Ahmanson-Lovelace Brain Mapping Center, Department of Neurology, University of California Los Angeles, Los Angeles, California, United States of America; University of Pennsylvania, United States of America

## Abstract

**Purpose:**

This study was aimed to experimentally and numerically investigate the feasibility of measuring cerebral white matter perfusion using pseudocontinuous arterial spin labeling (PCASL) 3T magnetic resonance imaging (MRI) at a relatively fine resolution to mitigate partial volume effect from gray matter.

**Materials and Methods:**

The Institutional Research Ethics Committee approved this study. On a clinical 3T MR system, ten healthy volunteers (5 females, 5 males, age = 28±3 years) were scanned after providing written informed consent. PCASL imaging was performed with varied combinations of labeling duration (τ = 1000, 1500, 2000, and 2500 ms) and post-labeling delay (PLD = 1000, 1400, 1800, and 2200 ms), at a spatial resolution (1.56x1.56x5 mm^3^) finer than commonly used (3.5x3.5 mm^2^, 5-8 mm in thickness). Computer simulations were performed to calculate the achievable perfusion-weighted signal-to-noise ratio at varied τ, PLD, and transit delay.

**Results:**

Based on experimental and numerical data, the optimal τ and PLD were found to be 2000 ms and 1500-1800 ms, respectively, yielding adequate SNR (~2) to support perfusion measurement in the majority (~60%) of white matter. The measurement variability was about 9% in a one-week interval. The measured white matter perfusion and perfusion ratio of gray matter to white matter were 15.8-27.5 ml/100ml/min and 1.8-4.0, respectively, depending on spatial resolution as well as the amount of deep white matter included.

**Conclusion:**

PCASL 3T MRI is able to measure perfusion in the majority of cerebral white matter at an adequate signal-to-noise ratio by using appropriate tagging duration and post-labeling delay. Although pixel-wise comparison may not be possible, region-of-interest based flow quantification is feasible.

## Introduction

Abundant studies have revealed a close relationship between cerebral perfusion (also referred to as cerebral blood flow, CBF) and brain function as well as physiology [1,2]. Abnormal CBF has been reported in a variety of neurological diseases and disorders [3-6]. While the majority of CBF related studies have focused on gray matter, aberrant white matter perfusion has been observed in schizophrenia [7], normal pressure hydrocephalus [8], and multiple sclerosis [9]. Although the physiological and pathophysiological mechanisms underlying these findings are not yet fully understood, reliable and quantitative measurement of CBF is desirable for its potential of facilitating clinical diagnosis/prognosis, treatment formulation, and neuroscience investigation.

Arterial spin labeling (ASL) magnetic resonance imaging (MRI) is a technique devised to measure perfusion free of administering exogenous contrast medium [10]. With ASL, the protons of arterial blood that feeds the area one wishes to measure perfusion from are magnetically labeled (or tagged) by radiofrequency (RF) pulses through inversion or saturation, and then serve as an endogenous tracer. Image acquisition follows after a post-labeling delay to allow for delivery of the tags. In most designs, a control image is also acquired in which no net magnetization perturbation is imposed upon the arterial blood. By referring to a proper biophysical model, the signal difference between the control and tag images can be quantified into perfusion in absolute units. One caveat of ASL is its inherently low signal-to-noise ratio (SNR) that depends on flow rate, labeling efficiency, and post-labeling delay during which the generated flow contrast persistently decreases because of longitudinal relaxation. In the brain, ASL has proven capable of providing reliable flow measurement in gray matter [11,12] whereas its feasibility in white matter is still under debate [13,14].

In comparison with gray matter, blood flow in white matter has been reported to be 1.6- to 4.6-fold lower [14-17] and it takes a longer transit time for the tags to travel from the labeling region to capillaries (a longer post-labeling delay is thus required), both of which compromise the SNR that is already low. A recent study [14] showed that measurement of white matter perfusion might be possible by using pseudocontinuous ASL (PCASL). PCASL is a relatively novel ASL technique [18] that employs a train of short RF pulses to emulate continuous labeling, amenable for body coil transmission and phased array reception, and whereby achieving the optimal balance between SNR and labeling efficiency as compared to conventional labeling methods [19].

In this study, we tried to optimize the labeling duration and post-labeling delay so as to distinguish flow-related signal of white matter from background noise using PCASL 3T MRI. Based on experimental results and numerical simulations, the feasibility and reliability of quantitatively measuring perfusion in cerebral white matter were investigated on a cohort of healthy volunteers.

## Materials and Methods

### MR Experiments

The Research Ethics Committee at National Taiwan University Hospital approved this study. Ten healthy volunteers (5 females, 5 males, age = 28±3 years) were recruited and all gave written informed consent before participation. MR imaging was performed on a 3T whole body scanner (Tim Trio, Siemens, Erlangen, Germany) using the body coil to transmit RF pulses and a 12-channel phased-array head coil to receive signals. The subject was placed supine and head first. Earplugs were given to shield acoustic noise. Foam pads were firmly wedged between head and coil for stabilization.

After scout scans, anatomic images were obtained using T_1_-weighted three-dimensional magnetization-prepared rapid gradient-echo imaging (TR = 2530 ms, TE = 2.27 ms, TI = 1100 ms, flip angle = 9°, voxel size = 1x1x1 mm^3^). A series of PCASL scans followed with varied labeling duration (τ = 1000, 1500, 2000, and 2500 ms) and post-labeling delay (PLD = 1000, 1400, 1800, and 2200 ms). A single-shot gradient-echo echo-planar sequence was used for data readout: TR = 5500 ms, TE = 18 ms, in-plane matrix = 128x128, GRAPPA acceleration factor = 2, 6/8 partial Fourier, 12 axial slices approximately centered at the level of corpus callosum and acquired sequentially in an inferiosuperior order, inter-slice gap = 1 mm in space and 60 ms in time, slice thickness = 5 mm, field-of-view = 200 mm (i.e., nominal voxel size = 1.56x1.56x5 mm^3^), 80 measurements (i.e., 40 pairs of control and tag images). The control/labeling plane was placed 80 mm below the center of the imaging volume. Each scan took 7 min and 20 sec. To calibrate labeling efficiency, two additional PCASL scans were carried out: τ = 1500 ms, PLD = 1000 ms, 10 measurements, phase offset of labeling pulses = 50° and 100°, respectively [12]. For coil sensitivity correction and flow quantification, two reference scans were appended (single-shot gradient-echo echo-planar sequence, TR = 15 s, TE = 18 ms, in-plane matrix = 64x64, one received with the phased-array coil and the other with the body coil). Coil sensitivity was estimated by taking the ratio of the two reference images. The total scan time was about two hours. On two subjects, PCASL imaging was also performed with the control/labeling plane placed 80 mm distally to assess residual signal perturbation from the control/labeling pulses.

To test measurement reproducibility, the above experiment (proximal control/labeling only) was repeated on eight of the subjects after one week, during the similar time of a day. None of these subjects reported notable changes in caffeine ingestion and sleep/stress condition between experiments. All these subjects self-reported to be non-habitual and non-heavy caffeine consumers (less than 400 mg per week over the past 6 months). They were asked to abstain from caffeine intake 24 hours before the experiment.

### Data Analysis

All complex data were reconstructed online into magnitude images, and then exported to a laptop computer for post-processing using Statistical Parametric Mapping, version 2 (SPM2; http://www.fil.ion.ucl.ac.uk/spm/) and custom designed programs under the environment of MATLAB (The MathWorks Inc., Natick, MA).

ASL images were corrected for head motion and spatially varying coil sensitivity, and then pair-wise subtracted to generate perfusion-weighted images (dM = control image – tag image). For each τ and PLD, dM’s were averaged and converted to quantitative CBF maps based on a published procedure with labeling efficiency calibration included [12]. For each subject, the anatomic images were segmented into gray matter, white matter, and cerebrospinal fluid (CSF), and then co-registered to the mean motion-corrected ASL images. Based on the probability maps SPM2 provided, binary masks were created for gray matter at a probability threshold of 0.95 and for white matter at probability thresholds of 0.95 and 0.99. Part of the frontal lobe was manually excluded because of unsatisfactory co-registration caused by excessive geometric distortion in echo-planar images. Subsequent analysis of signal intensity and perfusion was performed within these masks unless otherwise noted. The mean (X) and standard deviation (SD) of ghost-free background were computed. Detectable voxels were defined as those with signal intensity larger than X+SD [13]. The mean intensity of dM was plotted against τ and PLD, respectively, to determine the optimal τ and PLD, based on which, perfusion-weighted SNR (SNR_dM_) was examined.

SNR_dM_ was also computed for spatially smoothed data. Two degrees of smoothing were created by using a three-dimensional Gaussian kernel with a full-width-half-maximum (FWHM) of 3 mm and 8 mm, respectively. While the former yielded an effective in-plane resolution of 3.19 mm to approximate the resolution commonly used in ASL imaging (~3.5 mm), the latter was the kernel size many previous studies had adopted in preprocessing for group-level data analysis. The effective resolution was computed as the ratio of the area under curve to the height, which for a Gaussian function can be related to FWHM by a factor of $0.5\sqrt{\pi /\ln(2)}$ (ln is the natural logarithm). Note that the effective resolution here did not take account of the point spread function [20] determined by intrinsic transverse relaxation, readout trajectory, and extrinsic degradation factors such as susceptibility and inhomogeneity of B_0_/B_1_ fields. To assess the partial volume effect caused by smoothing (or at coarser resolutions), we applied the same kernels to the abovementioned probability maps of gray matter and white matter. For voxels containing gray matter or white matter at a probability above 0.95, we examined their probability histograms both before and after smoothing. Partial volume effect was deemed absent or negligible in a voxel whose post-smoothing probability remained above 0.95.

To generate group maps of SNR_dM_, all datasets were pooled to create templates for both anatomic and echo-planar images. Each subject’s images were normalized to the templates using affine transformation. Binary masks of gray matter and white matter were generated by applying the aforementioned procedure to the anatomic template. The mentioned kernels were then applied to the group SNR_dM_ and masks to assess the effect of spatial smoothing. 

Measurement reproducibility was evaluated in terms of the within-subject coefficient of variation (wsCV), the square root of mean square of per-subject CV.

### Computer Simulation

Theoretical SNR of PCASL measurement was calculated using computer simulations. When PCASL images are acquired at time t after the start of control/labeling pulses, the intensity of dM can be formulated as [21] 

$$dM(t)=2\cdot \alpha \cdot M_{0b}\cdot f\cdot g\left(t,\tau ,\delta ,T_{ex},T_{1b},T_{1t}\right)$$ (1)

in which M_0b_ is the fully relaxed longitudinal magnetization of arterial blood, α and f are labeling efficiency and blood flow, respectively. Function g accounts for the temporal evolution dictated by labeling duration (τ), transit time (δ), exchange time (T_ex_), longitudinal relaxation time constant of tissue (T_1t_) and arterial blood (T_1b_). T_ex_ is the time period before a tag enters the extravascular-extracellular space after its arrival at the capillary bed. Note that post-labeling delay (PLD) is implicitly included through t = τ + PLD. Based on the framework described by van Gelderen et al [13], the SNR of dM can be related to the SNR of M_0b_:

$$SNR_{dM}=2\cdot \alpha \cdot f\cdot g\left(t,\tau ,\delta ,T_{ex},T_{1b},T_{1t}\right)\cdot \sqrt{0.5}\cdot \sqrt{N}\cdot SNR_{M_{0b}}$$ (2)

where the square root of 0.5 is the penalty of using pair-wise subtraction to generate dM images, N is the number of dM images averaged (i.e., the number of control/tag pairs in our case). To estimate SNR_M0b_, we selected from the reference scan a slice that contained the lateral ventricles with at least 5 contiguous voxels that according to aforementioned anatomic masks were classified as CSF at a probability threshold of 0.95. The SNR of CSF (SNR_M0c_) was computed by taking the ratio of the average signal intensity of those CSF voxels to the standard deviation of the signal intensity within a region of interest placed at the background where no ghost or structured artifacts existed. SNR_M0c_ was then related to SNR_M0b_ by


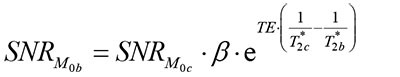
(3)

where β was the proton density ratio of arterial blood to CSF, T_2b_* and T_2c_* were the transverse relaxation time constant of arterial blood and CSF, respectively. The parameters used in the simulation are summarized in Table 1. The effect of different transverse relaxation rates between blood and tissue [22] was not included in our simulation.

**Table 1:** Summary of the parameters used in computer simulations.

**Parameter**	**Value**	**Unit**
α	0.85	
f	WM = 20, GM = 60	ml/100ml/min
N	40	
β	0.88	
T_ex_	100	ms
T_1t_	WM = 1000 ms, GM = 1330 ms	ms
T_1b_	1660	ms
T_2b_*	80	ms
T_2C_*	250	ms

The listed parameters are labeling efficiency (α), blood flow (f), pair number of control/tag images (N), exchange time (T_ex_), longitudinal relaxation time constant of tissue (T_1t_) and arterial blood (T_1b_), transverse relaxation time constant of arterial blood (T_2b_*) and CSF (T2C*), proton density ratio of arterial blood to CSF (β). CSF = cerebrospinal fluid. GM = gray matter. WM = white matter.

## Results

**Figure 1:**
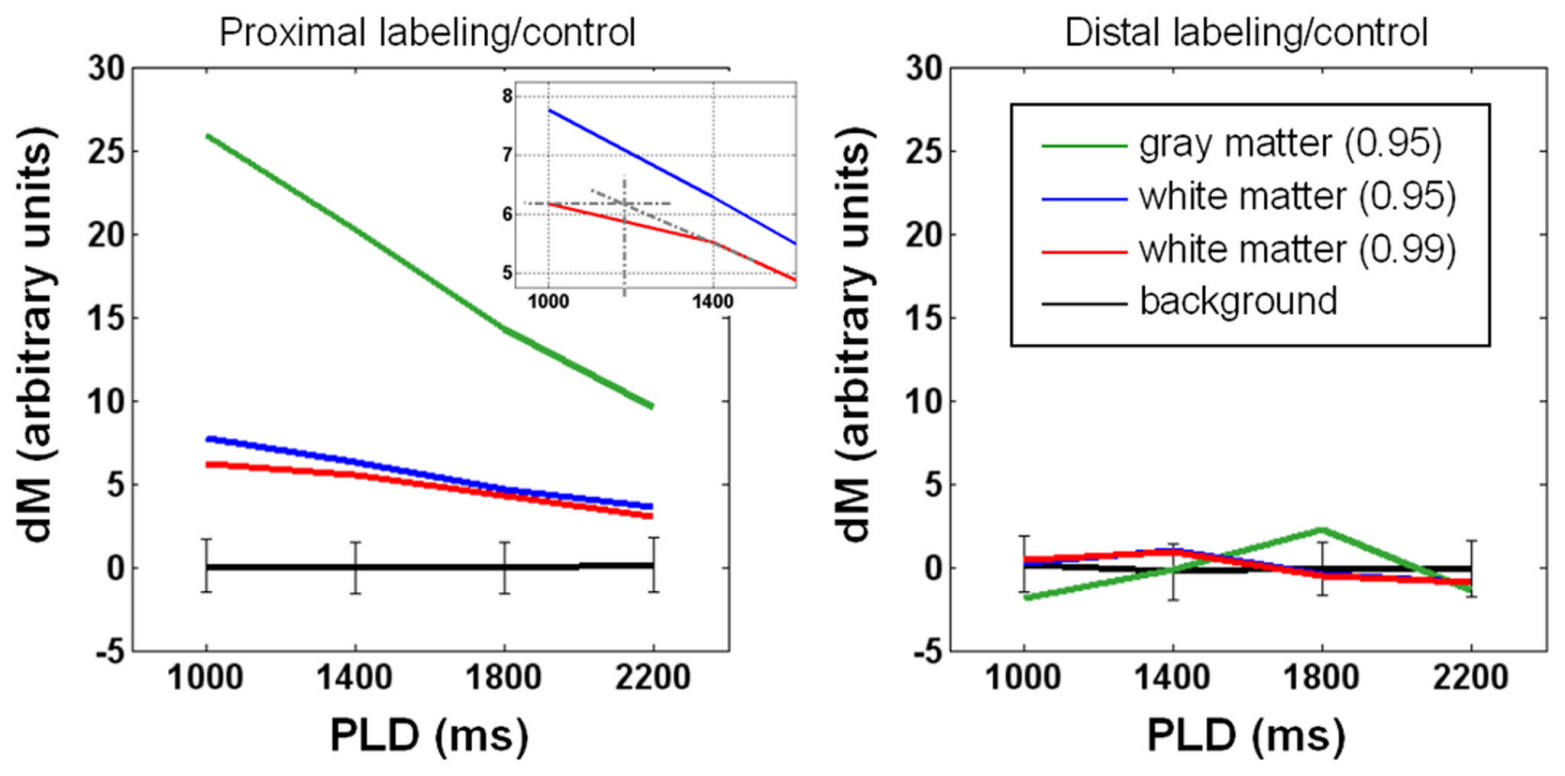
Dependence of perfusion-weighted signal intensity (dM) on post-labeling delay (PLD). Tagging duration = 1500 ms. Data is obtained from the 6th slice of a representative subject. Regions of interest are determined at a probability of 0.95 for gray matter (green lines), and both 0.95 and 0.99 for white matter (blue and red, respectively). The inset in the left panel suggests a transit delay of ~1200 ms. In both panels, background signals are zero on average with standard deviation (i.e., noise) shown in error bars. The right panel shows that labeling/control pulses cause negligible flow-irrelevant signal perturbation.

**Figure 2:**
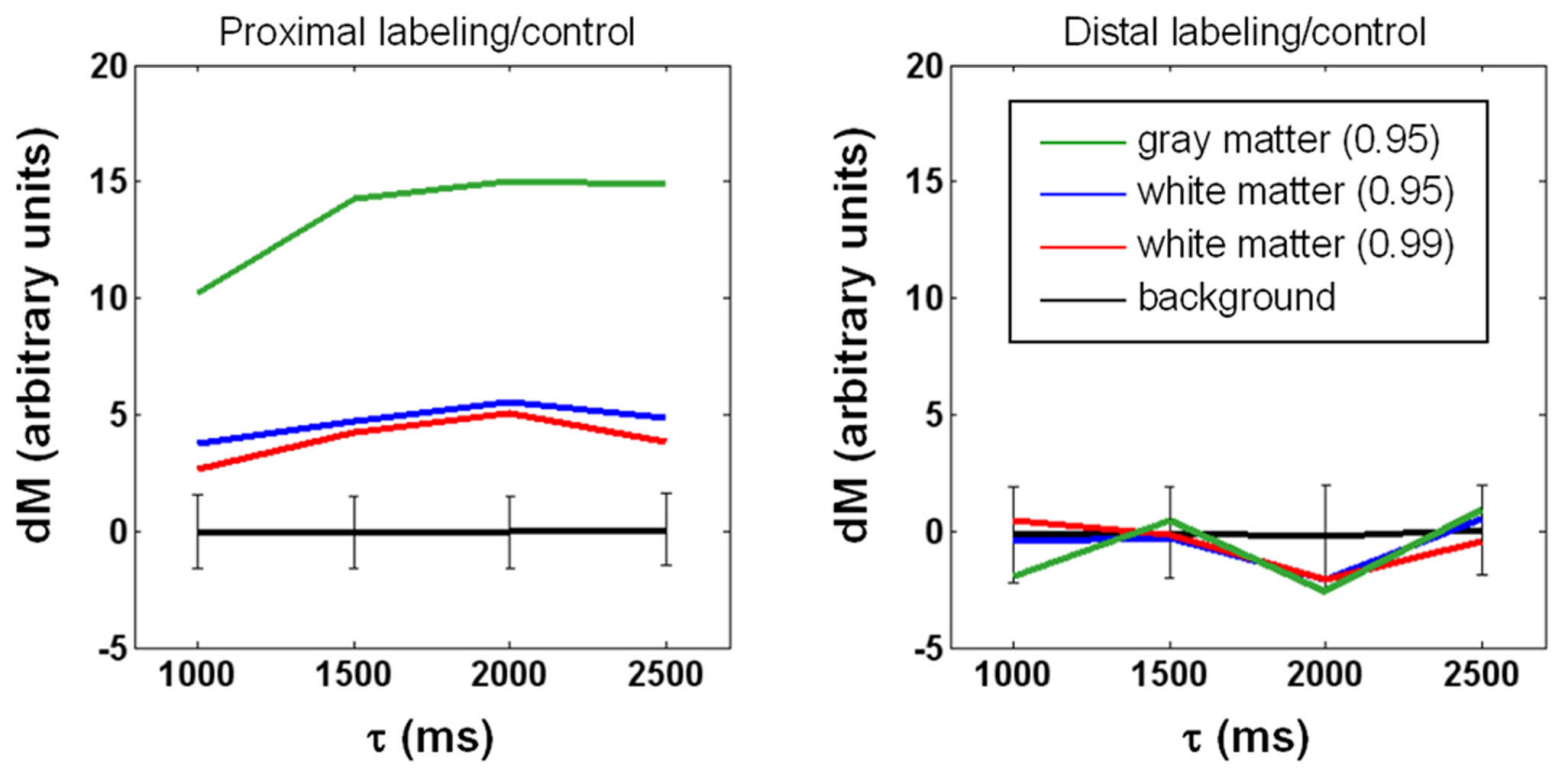
Dependence of perfusion-weighted signal intensity (dM) on tagging duration (τ). Post-labeling delay = 1800 ms. Data is obtained from the 6th slice of the subject in Figure 1. Regions of interest are determined at a probability of 0.95 for gray matter (green lines), and both 0.95 and 0.99 for white matter (blue and red, respectively). In both panels, background signals are zero on average with standard deviation (i.e., noise) shown in error bars. The right panel indicates that labeling/control pulses cause negligible flow-irrelevant signal perturbation.

**Figure 3:**
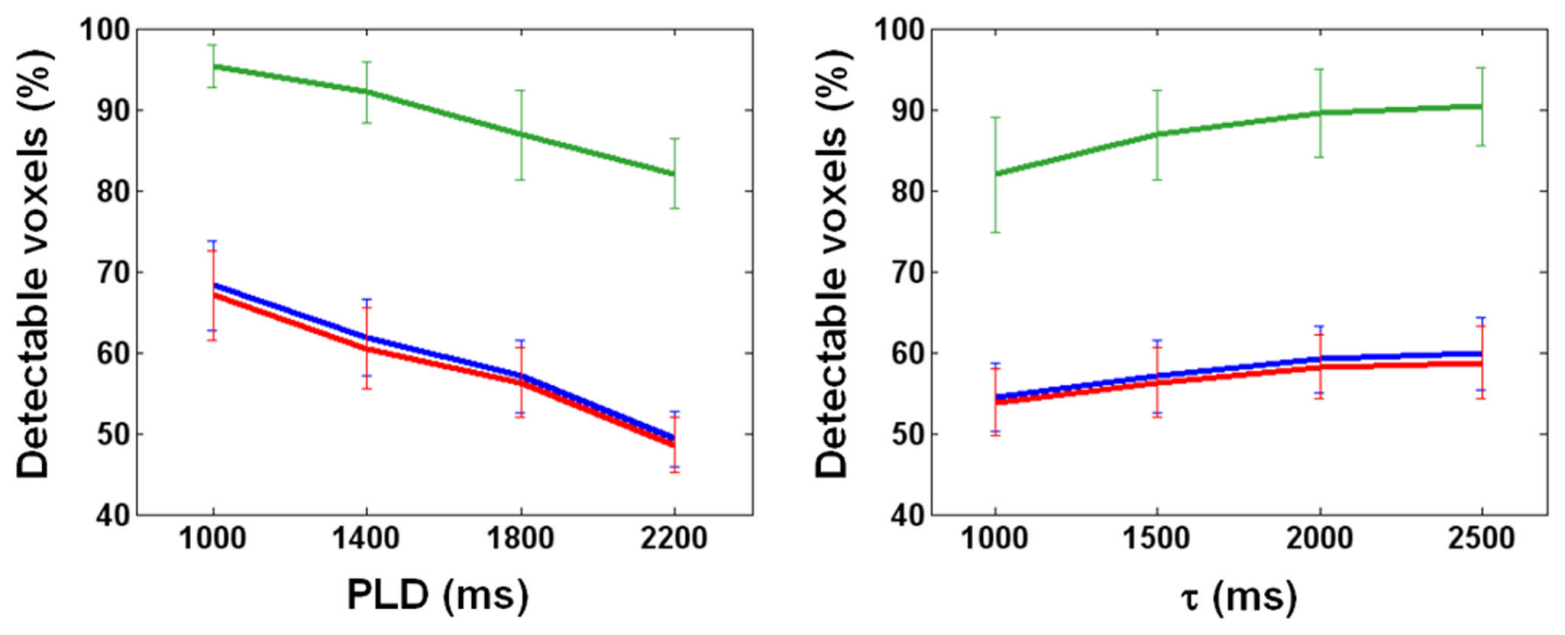
Percentage of voxels detectable at varying post-labeling delay PLD (left panel, tagging duration = 1500 ms) and tagging duration τ (right panel, PLD = 1800 ms). Error bars indicate the standard deviation across 10 subjects.

**Figure 4:**
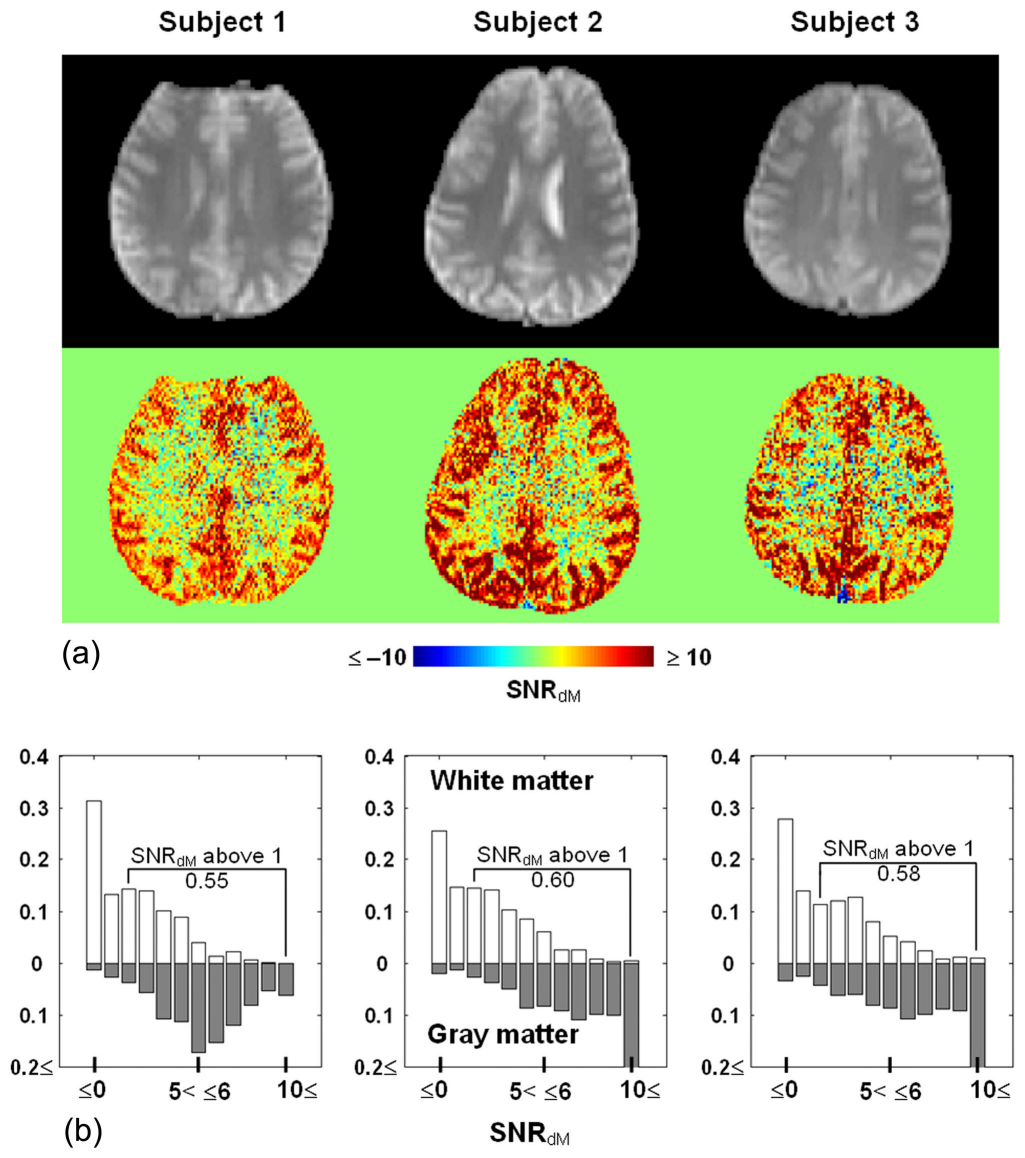
Maps (a) and histograms (b) of perfusion-weighted signal to noise ratio (SNR_dM_) obtained in three representative subjects (slice 6). Echo-planar images at the same location are shown for anatomical reference (upper row). In (b), white bars = white matter, gray bars = gray matter. The proportion of white matter voxels with SNR_dM_ above unity is 0.55, 0.60, and 0.58, respectively.

In Figure 1, the data was obtained from the sixth slice of a representative subject, showing that when PLD increases, with τ fixed at 1500 ms, dM monotonically decreases in both white matter and gray matter. Given the inferiosuperior acquisition order and 60-ms inter-slice gap, the actual PLD shown here is the nominal value plus 300 ms. As such, the transit time is about 1200+300 ms for white matter as the inset suggests, while the transit time of gray matter, previously reported to vary between 600 ms and 1200 ms [23,24], is apparently shorter than 1000+300 ms. By contrast, no clear trend exists under the distal control/labeling condition. Also obtained from the sixth slice of the same subject in Figure 1, data in Figure 2 shows that with PLD fixed at 1800 ms, dM increases with τ and reaches a plateau when τ is around 2000 ms, whereas no τ dependence is observed in dM intensity when control/labeling pulses are applied distally. The pattern of dM varying with τ and PLD was found largely preserved across slices and subjects. We therefore computed for each subject the percentage of detectable voxels (see Materials and Methods for definition). The percentage was averaged across subjects and shown in Figure 3. Ideally, PLD should be no shorter than the physiological transit time but as short as possible to avoid unnecessary SNR decrease. According to Figures 1 and 3, the optimal PLD is 1500-1800 ms for more than half of white matter to be detected. Figures 2 and 3 together indicate that the optimal τ is 2000 ms as further extension provides limited benefit to dM signals and the number of detectable voxels. As such, the following results of SNR_dM_ and smoothing analysis will be based on the data acquired with τ = 2000 ms and PLD = 1800 ms, in which approximately 60% of white matter and 90% of gray matter are detectable. Figure 4 shows the maps and histograms of SNR_dM_ obtained in three representative subjects.

**Figure 5:**
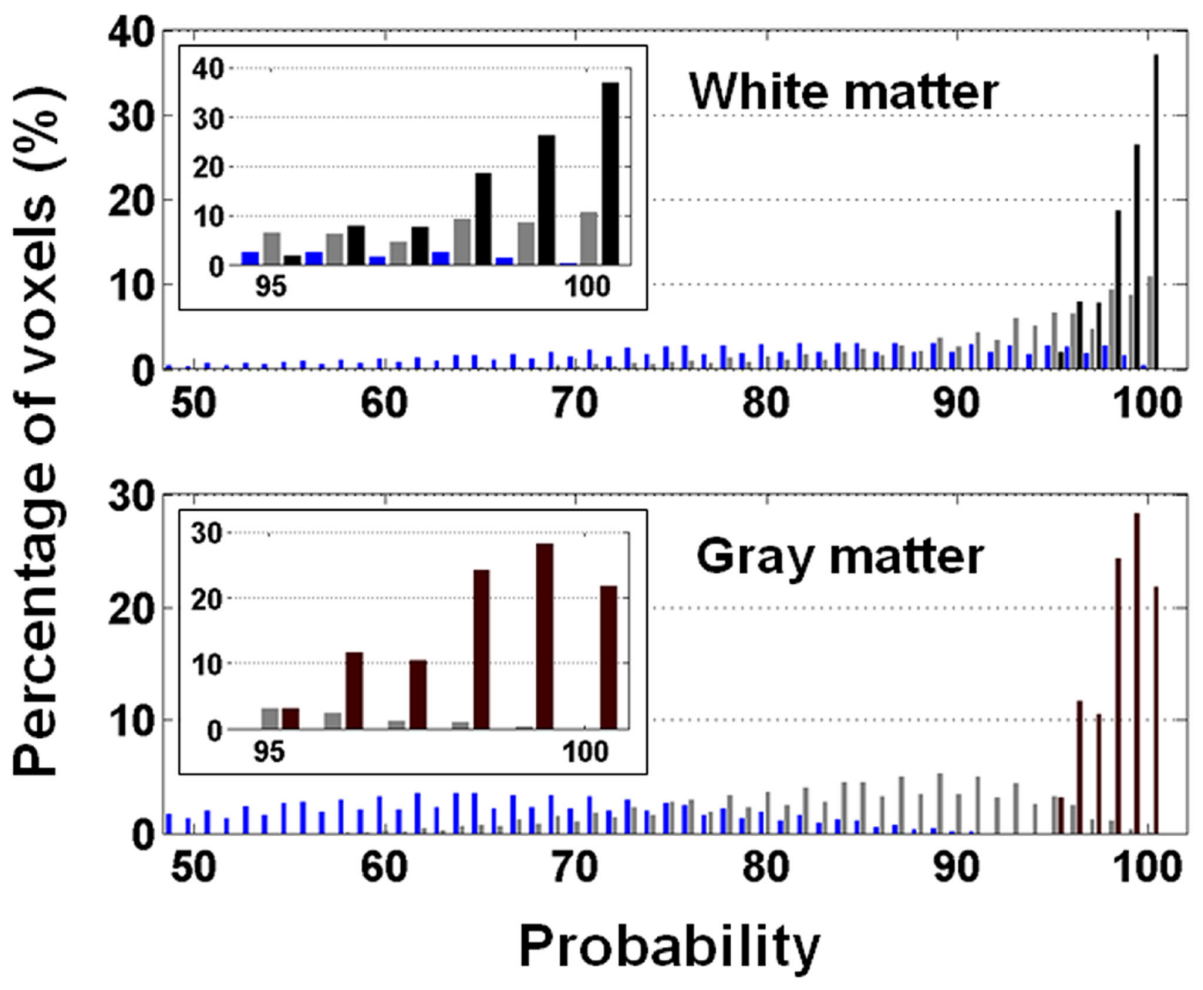
Histogram of tissue probability. Shown in black bars is the original histogram that only contains pixels with tissue probability above 0.95 (upper panel = white matter, lower panel = gray matter). After spatial smoothing, the probability histogram of these voxels is overlaid on the original histogram for comparison. A three-dimensional Gaussian-shaped kernel was used for smoothing (full-width-half- maximum = 3 mm as shown in gray bars and 8 mm as shown in blue bars). The insets are the blowup view of tissue probability ≥ 0.95.

Figure 5 demonstrates the partial volume effect in the presence of spatial smoothing or at coarser resolutions (data from a representative subject). The effect should be subject-independent and indeed we found it very similar across subjects. The volume fraction of white matter and gray matter was estimated based on their probability maps. Here we had used a threshold of 0.95 to extract from the un-smoothed data a subset of voxels “free of partial volume” (shown in black bars). After smoothing, the probability distribution of the subset is broadened and shifted to lower values. When the kernel is 3 mm in FWHM, 43% of the original white matter voxels and merely 7% of the original gray matter voxels are free of partial volume (shown in gray bars). The percentage further drops to 11 for white matter and less than 0.1 for gray matter when the kernel’s FWHM is increased to 8mm (shown in blue bars). 

**Figure 6:**
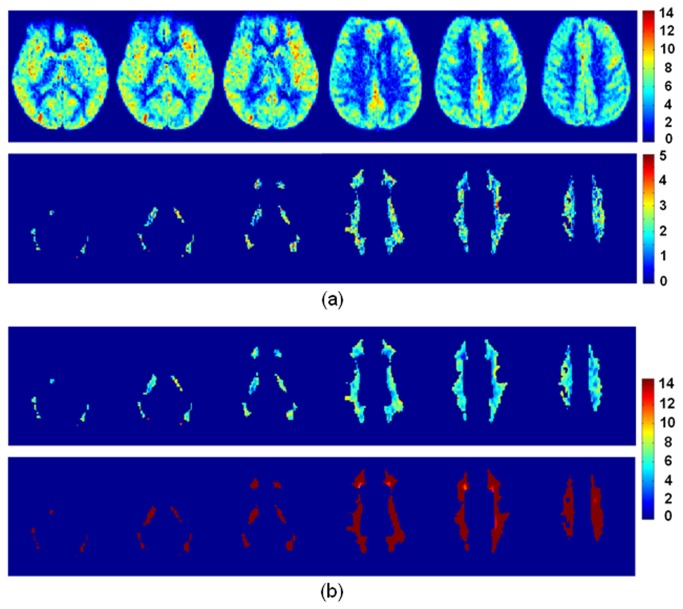
Maps of perfusion-weighted signal to noise ratio (SNR_dM_) averaged across 10 subjects. Six slices are shown. (a) SNR_dM_ is noticeably higher in gray matter than in white matter. To better display how SNR_dM_ is distributed in white matter, gray matter is masked out in the lower panel and the color scale is adjusted. (b) SNR_dM_ in white matter after spatially smoothed by a three-dimensional Gaussian-shaped kernel (full-width-half- maximum = 3 mm for the upper panel and 8 mm for the lower panel). Note that a different color scale is used to accommodate the increase of SNR_dM_.

Shown in Figure 6 are representative slices of group SNR_dM_ maps (N = 10). As expected, SNR_dM_ is noticeably higher in gray matter than in white matter (Figure 6a). To better highlight the spatial distribution of SNR_dM_ in white matter (probability threshold = 0.95), color scale was adjusted after removal of gray matter. Indeed, SNR_dM_ is above unity in most white matter regions, and can be enhanced by spatial smoothing at the expense of blurring (Figure 6b).

Table 2 summarizes the SNR_dM_ and CBF measured from data with and without spatial smoothing. Values extracted with pre- and post-smoothing masks are both shown for white matter, but only pre-smoothing masks for gray matter in consideration of the prominent partial volume effect. The post-smoothing masks serve as more stringent selection of white matter, presumably the deep white matter where transit time is most prolonged.

**Table 2:** Summary of perfusion-weighted signal to noise ratio (SNR_dM_) and CBF calculated from data before and after application of spatial smoothing.

	**SNR_dM_**		**CBF (SNR_dM_ > 1)**	
**Degree of smoothing**	**WM**	**GM**	**WM**	**GM**
w/o smoothing	1.9 +/– 0.4	7.3 +/– 2.4	27.5 +/– 1.6	63.9 +/– 1.8
FWHM = 3 mm	6.2 +/– 1.3 (4.8 +/– 0.9)	19.6 +/– 6.8	24.4 +/– 1.6 (18.6 +/– 1.6)	53.8 +/– 3.5
FWHM = 8 mm	24.0 +/– 6.3 (14.2 +/– 3.6)	46.3 +/– 16.2	25.3 +/– 3.5 (15.8 +/– 3.5)	46.0 +/– 3.9

Pre-smoothing masks were applied to both white matter (WM) and gray matter (GM). Post-smoothing masks were also applied to WM with the results shown in parentheses. CBF is in units of ml/100ml/min. Data from 10 subjects are averaged and presented as mean +/- standard deviation.

Table 3 summarizes the wsCV of CBF measurements in a one-week interval. Spatial smoothing causes little difference in measurement variability in both white matter and gray matter.

**Table 3:** Reproducibility of CBF measurement in a one-week interval (N = 8).

**Degree of smoothing**	**WM wsCV CBF**	**GM wsCV CBF**
w/o smoothing	8.4%	7.5%
FWHM = 3 mm	9.0%	7.0%
FWHM = 8 mm	9.2%	7.0%

Calculation was performed based on the voxels where signal to noise ratio was above 1. GM = gray matter; WM = white matter; wsCV = within-subject coefficient of variation.

**Figure 7:**
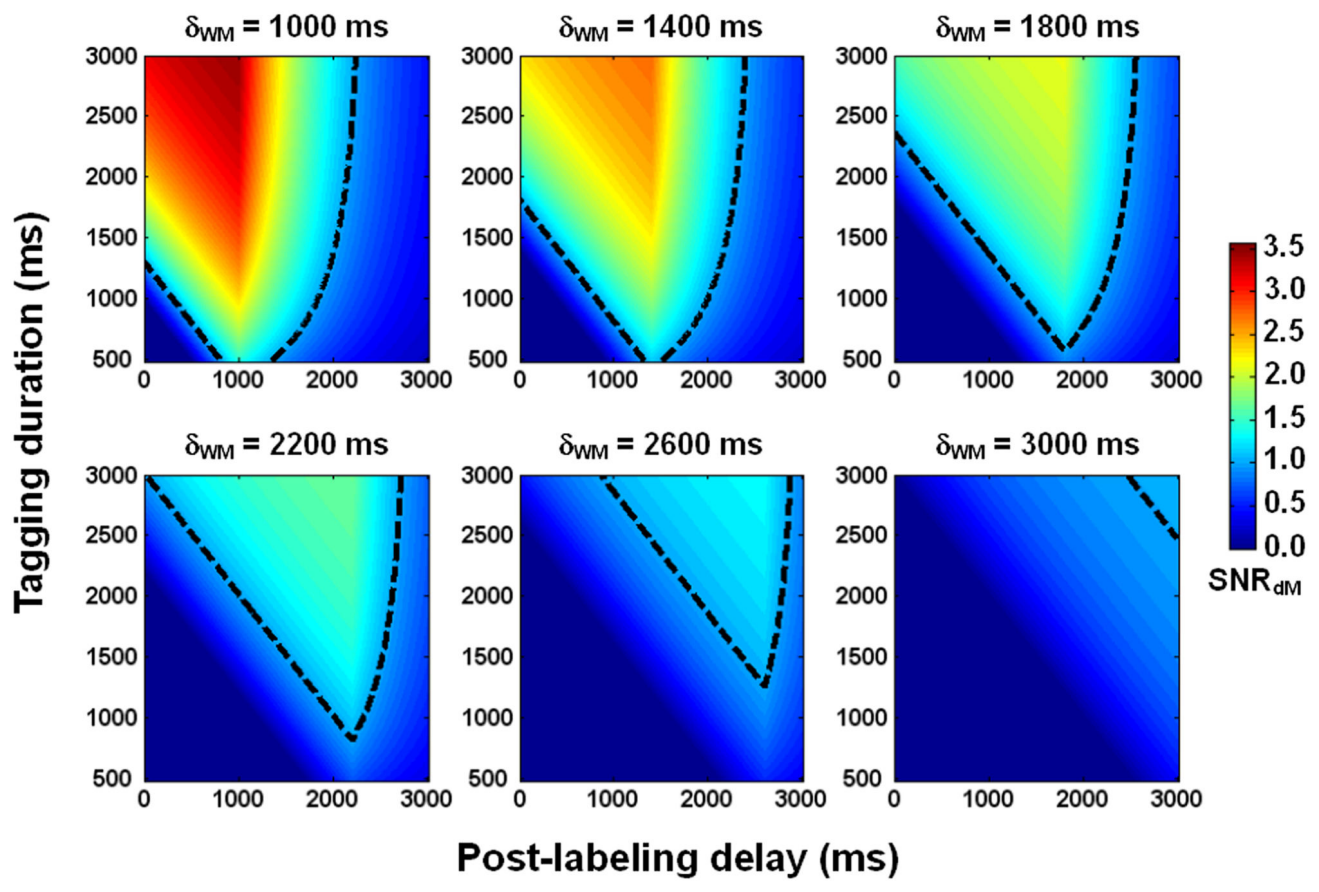
Simulated dependence of perfusion-weighted signal to noise ratio (SNR_dM_) on tagging duration and post-labeling delay in white matter. Transit delay (δ_WM_) is increased from 1000 ms to 3000 ms, with a step of 400 ms. The dashed lines indicate the contours where SNR_dM_ = 1.

Figure 7 displays the numerically generated dependence of SNR_dM_ on τ and PLD in white matter, suggesting that white matter perfusion is measurable using PCASL 3T MRI through appropriate choice of τ and PLD. In our experiment, these two parameters were landed in the regime where SNR_dM_ is predicted to be above unity for a wide range of transit times.

## Discussion

The applicability of ASL in measuring perfusion of cerebral white matter has been pending mainly due to the inherently low flow rate and prolonged transit time in white matter. In this study, we have systematically investigated the perfusion-weighted signal-to-noise ratio (SNR_dM_) obtainable in white matter using PCASL 3T MRI. Experimental data indicate that it is possible to measure perfusion in the majority of white matter at an adequate SNR level by using appropriate tagging duration (τ, or bolus width) and post-labeling delay (PLD).

**Figure 8:**
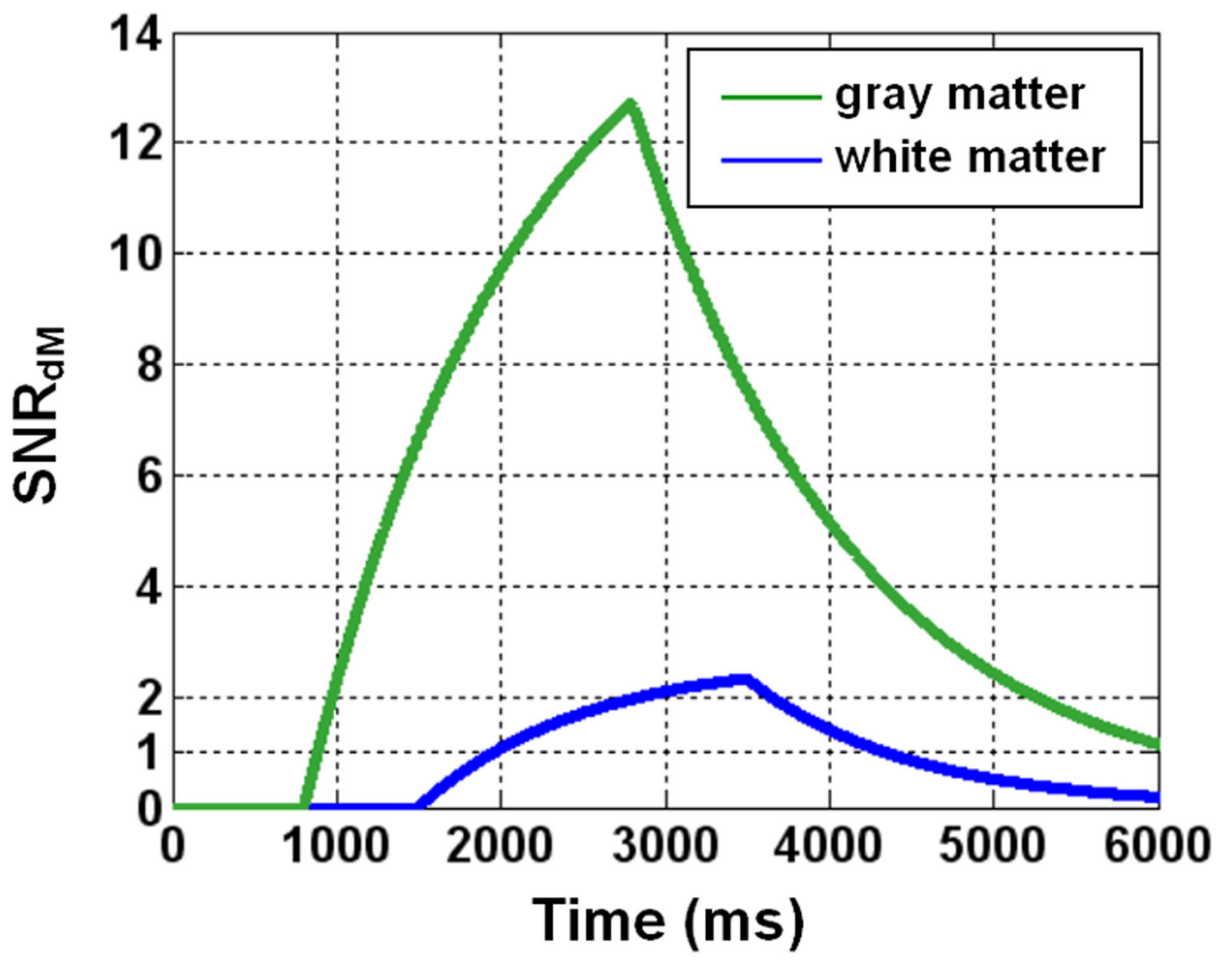
Simulated temporal evolution of perfusion-weighted signal to noise ratio (SNR_dM_). Tagging duration = 2000 ms, transit delay = 800 ms for gray matter and 1500 ms for white matter.

In general, SNR_dM_ increases with τ and decreases with PLD (Figures 1-3). The optimal values derived experimentally are 2000 ms for τ and 1500-1800 ms for PLD, reasonably agreeing with numerical simulations (Figure 7) that further indicate that the optimal values are dependent on transit time. For our imaging setting (particularly the position of center slice, two-dimensional readout, inferiosuperior acquisition with a 60-ms inter-slice gap, and voxel size = 1.56x1.56x5 mm^3^), a bolus of 2000 ms provides sufficient SNR_dM_ while a nominal PLD of 1500-1800 ms (the actual PLD for the most proximal slice) is adequate to ensure the arrival of most tags in all slices without too much compromise in SNR_dM_. Figure 8 shows the theoretical SNR_dM_ evolution based on our imaging parameter (τ = 2000 ms) and experimental results (transit time = 1500 ms in white matter) and other assumptions listed in Table 1. Ideally, data should be acquired no earlier than the SNR_dM_ peak of white matter (to avoid confounding arterial signals) and before it decays below 1. It is noted that further extension of PLD may be needed when prolonged transit time is expected, for example in elderly population and patients with ischemia. In the extreme case, ASL will not be reliable in measuring white matter perfusion as no combination of τ and PLD supports adequate SNR_dM_ (for example, the bottom right panel in Figure 7). It should also be noted that the SNR_dM_ of gray matter will be compromised when one optimizes τ and PLD for white matter.

Partial volume effect has been recognized as a confounder in ASL measurement of white matter perfusion [13,14]. As justifiable as the concern is, we found that gray matter is no less susceptible to partial volume effect than white matter even though many studies have reported reliable measurement and satisfactory SNR_dM_ in gray matter. Let’s consider the resolution used in our imaging as the reference standard (1.56x1.56x5 mm^3^). Figure 5 shows that spatial smoothing with a kernel of 3-mm FWHM brings in partial volume effect to 93% of the gray matter. The kernel size corresponds to effective in-plane resolution of 3.19 mm that has been adopted in many published ASL studies. The spread of probability histogram leads to 12% flow underestimation on average (assuming that CBF = 60, 20, and 0 ml/100ml/min for gray matter, white matter, and CSF, respectively). When the kernel is increased to 8 mm in FWHM (common preprocessing for group analysis), nearly all gray matter is affected by partial volume effect and the underestimation of average flow can be up to 35%. Meanwhile, partial volume affects 57% and 89% of white matter with a kernel size of 3 mm and 8 mm, respectively, and the average flow is overestimated by 18% and 35%, respectively. The ultimate solution to partial volume effect is to further increase the resolution, which however, may not be possible due to hardware restriction and/or SNR limit. Alternatively, partial volume effect may be estimated and corrected for using a regression method [16]. The method, however, introduces inherent spatial blurring due to the assumptions that tissue magnetization and perfusion are constant within a predefined kernel. Any included voxels with SNR_dM_ below 1 may bring in additional errors.

The perfusion ratio of gray matter to white matter varies widely, roughly from 1.6 to 4.6 [14-17], in literature. Different measurement techniques, processing procedures, and quantitative models may all contribute to the variation. Another cause manifested in our study is spatial resolution. As shown in Table 2, the ratio is 2.3 for non-smoothed data, 2.2 after 3-mm smoothing, and 1.8 after 8-mm smoothing. As mentioned, partial volume effect affects both gray matter and white matter. Hence, gray matter perfusion obtained without smoothing should be closest to the true value while spatial smoothing leads to noticeable underestimation. In white matter, spatial smoothing brings in two competing effects. While erroneous inclusion of gray matter overestimates white matter perfusion, spatial smoothing may increase SNR such that some deep white matter that is excluded at first due to low SNR is now included and brings down the average (given that flow is lower in deep white matter). In this sense, the perfusion extracted with the post-smoothing masks (values shown in parentheses) can be deemed as the more conservative but less adulterated area of white matter. Specifically, the 8mm-smoothing mask consists of the largest proportion of deep white matter than other masks. As such, the perfusion ratio of gray matter to white matter is 2.2, 3.4, and 4.0, increasing with the proportion of deep white matter.

In this study, we used a resolution finer than usually reported (1.56x1.56x5 vs. ~3.5x.3.5 mm^2^ and 5-8 mm in thickness) to mitigate partial volume at the expense of SNR_dM_ which was around 2, increased but still below 10 after the images were smoothed to an effective in-plane resolution of 3.19 mm. Therefore, pixel-wise comparison should be carried out with caution whenever needed. Nonetheless, our data reveal that reproducible perfusion measurement can be obtained in the majority of cerebral white matter within 10 min, with correction of labeling efficiency and coil sensitivity included. These areas include semioval center, corona radiata, and internal capsule (Figure 6). It is possible to further improve SNR by incorporating background suppression. With background suppression [25], additional (usually two to four) inversion pulses are inserted between labeling and excitation pulses with their timing properly adjusted such that at the time of excitation the longitudinal magnetization of static tissues is near zero whereas the flow contrast is preserved. Background suppression ideally works for three-dimensional data acquisition but yields varied degrees of suppression between slices for two-dimensional acquisition (less effective for later acquired slices) and thus may bring in quantitative errors. In addition, the nonideal inversion efficiency caused by field inhomogeneity may confound quantification [26]. As a remedy, a pre-scan may be required to estimate the spatial distribution of the inversion efficiency of background suppression pulses. Another option is to use a phased array of more channels for signal reception. If time allows, more measurements can also be collected for average. 

Although ASL-measured perfusion values in cerebral white matter have been reported in literature, the measurement reliability has not been systematically addressed until two recent studies [13,14]. Based on pulsed ASL, van Gelderen et al [13] theoretically analyzed the SNR and partial volume effect, and concluded that white matter perfusion cannot be reliably measured. Based on PCASL and background suppression, van Osch et al [14] experimentally demonstrated the possibility of perfusion measurement in white matter, using a τ of 1650 ms in conjunction with a PLD of 1525 ms without optimization. In our study, a spatial resolution (1.56x1.56x5 mm^3^) finer than that in van Osch et al’ work (3x3x7 mm^3^) was adopted to reduce partial volume effect whereas background suppression was not included to avoid undesirable variability in inversion efficiency. Experimental optimization of PLD and τ were then carried out, followed by quantitative assessment of partial volume effect at the spatial resolutions that have been commonly used in ASL imaging. Finally, numerically calculated SNR revealed agreement with experimental data.

A few limitations exist in this study. Firstly, the presented optimal τ and PLD were only applicable to our imaging setting which however should be easy to adopt. Secondly, as Figure 7 suggests, the optimal τ and PLD will deviate from the presented values when transit time is elongated, especially beyond 2200 ms. Further investigation is needed in elderly population and patients with ischemia. Thirdly, we empirically increased the in-plane resolution rather than providing a theoretical limit of resolution with regard to SNR. Geometry factor related noise amplification had not been accounted for in our data analysis. It is worth noting that in this study, image coregistration and normalization had been performed using SPM2 with which the presence of atrophy and ventricular hypertrophy could lead to misregistration in cortical sulci. While this should cause little or no errors to our results in healthy volunteers, more sophisticated methods (e.g., DARTEL [27] in SPM version 8) will be required to process images obtained from patients or elderly population. Lastly, tissue segmentation can be improved by including multi-modal images (e.g., T2- and/or diffusion-weighted images), especially when anatomic variations increase as a result of disease or aging. 

In conclusion, we have experimentally and theoretically shown that PCASL 3T MRI is able to measure perfusion in the majority of cerebral white matter at an adequate SNR level by using appropriate tagging duration and post-labeling delay. The measured white matter perfusion is 15.8-27.5 ml/100ml/min and the perfusion ratio of gray matter to white matter is 1.8-4.0, both of which are dependent on spatial resolution and the amount of deep white matter included. By using the optimal labeling duration (2000 ms) and post-labeling delay (nominal value = 1500-1800 ms), the measurement variability is about 9% in a one-week interval. The scan time is within 10 min with required calibration/correction scans included, which is reasonable for clinical routines and makes possible physiological/functional investigation of white matter.
